# Decomposing differences in depressive symptoms between older rural-to-urban migrant workers and their counterparts in mainland China

**DOI:** 10.1186/s12889-020-09374-1

**Published:** 2020-09-23

**Authors:** Wei Yang, Dan Li, Jianmin Gao, Xiaojuan Zhou, Fuzhen Li

**Affiliations:** 1 Xi’an Jiaotong University Health Science Center, Xi’an, Shaanxi PR China; 2 Department of Public health, Central Hospital of Shangluo, Shangluo, Shaanxi PR China; 3 School of Public Policy and Administration, Xi’an Jiaotong University, Xi’an, Shaanxi PR China; 4 Department of Anesthesiology, Northwest Women and children’s Hospital affiliated to Xi’an Jiaotong University, Xi’an, Shaanxi PR China; 5 Department of Infectious Diseases, Central Hospital of Shangluo, Xi’an, Shaanxi PR China

**Keywords:** Depressive symptoms, Older rural-to-urban migrant workers, The social-ecological model, Fairlie’s decomposition

## Abstract

**Background:**

There has been an increase in older rural-to-urban migrant workers (aged 50 and above) in mainland China, little known about their depressive symptoms. The aim of this study was to identify depressive symptoms among older rural-to-urban migrant workers, as well as explored the factors leading to differences in depressive symptoms between older rural-to-urban migrant workers and their rural counterparts (older rural dwellers) and urban counterparts (older urban residents) in mainland China. The results provided a comprehensive understanding of the depressive symptoms of older rural-to-urban migrant workers, and had great significance for improving the depressive symptoms for this vulnerable group.

**Methods:**

Data were derived from the China Health and Retirement Longitudinal Study (CHARLS) conducted in 2015, and coarsened exact matching (CEM) method was employed to control confounding factors. This study employed a Chinese version 10-item short form of the Center for Epidemiologic Studies-Depression Scale (CES-D 10) to measure depressive symptoms, and used the Social-Ecological Model as a framework to explore influential factors related to depressive symptoms. Specifically, the approach of Fairlie’s decomposition was used to parse out differences into observed and unobserved components.

**Results:**

After matching, our findings indicated that the prevalence of depressive symptoms in older rural-to-urban migrant workers was lower than older rural dwellers; and the prevalence of depressive symptoms in older rural-to-urban migrant workers was higher than older urban residents. Fairlie’s decomposition analysis indicated that type of in-house shower, sleeping time at night and ill in the last month were proved to be major contributors to the differences in depressive symptoms between older rural-to-urban migrant workers and older rural dwellers; self-reported health and sleeping time at night were proved to be major contributors to the differences in depressive symptoms between older rural-to-urban migrant workers and older urban residents.

**Conclusions:**

Differences in depressive symptoms between older rural-to-urban migrant workers and their rural and urban counterparts did exist. Our findings contributed to a more reliable understanding in depressive symptoms among older rural-to-urban migrant workers. Our findings would be of referential significance for improving older rural-to-urban migrant workers’ depressive symptoms.

## Background

There has been a dramatic increase in rural-to-urban migrant workers in mainland since the 90’s of last century. At the same time, a number of rural-to-urban migrant workers are facing the situation of aging, and they are developing older rural-to-urban migrant workers (aged 50 and above), as National Bureau of Statistics (NBC) mentioned [1]. According to the NBC, on a national scale, the proportion of older rural-to-urban migrant workers in mainland China reached approximately 17.9% of the total rural-to-urban migrant workers in mainland China in 2015 [1]. Older rural-to-urban migrant workers have the dual characteristics of the rural-to-urban migrant workers and the elderly, which means they have more prominent health risks for depressive symptoms. Depressive symptoms is a notable contributor to the global economic burden of diseases worldwide. Psychological and neurological disorders are responsible for about 20% of the overall disease burden in mainland China in 2015 [2]. In 2015, psychological and neurological disorders are responsible for about 20% of the overall disease burden in mainland China [2]. China is a great country with the largest rural-to-urban migrant workers population in the world. The depressive symptoms problem-solving of this specific group is of great significance to achieve national health in mainland China.

Evaluating the mental health differences between older rural-to-urban migrant workers and their rural counterparts and their urban counterparts is essential for the mental health plans proposed to benefit older rural-to-urban migrant workers in mainland China. Mixed, sometimes contradictory mechanisms through which migration may potentially influence the mental health have been presented. However, there are still no consistency conclusions. On one hand, migration may cause disadvantages in many aspects, which had negative implications for rural-to-urban migrant workers’ depressive symptoms. Much research had demonstrated that migration was associated with an increased risk for poor mental health [3, 4]. Older rural-to-urban migrant workers were hindered by the lack of social capital, low-wage “3D” (dirty, dangerous and demanding) jobs, the strong mobility of work and the great intensity of work [5–7], which made older rural-to-urban migrant workers at high risk for depressive symptoms. Therefore, older rural-to-urban migrant workers in mainland China would be more likely to report relatively worse mental health status than those who were not experiencing the migration. On the other hand, extensive studies [8, 9] suggested that rural-to-urban migrant workers, who were likely to be a “selected” population with good health pre-migration, were generally healthier than the resident population at the origin and destination. This is often referred to as the “healthy migrant phenomenon” [8]. The “healthy migrant effect” isn’t exactly set in stone. There are some contentions with this theory on whether or not it exists to the extent that some of the literature states that it does [8].

Following the accelerated speed of population aging, older rural-to-urban migrant workers’ depressive symptoms should be taken seriously. Previous studies [10, 11] had discussed the depressive symptoms of age-heterogeneous group by comparing the new-generation rural-to-urban migrant workers (those born after 1980) and the old-generation rural-to-urban migrant workers (those born before 1980) in mainland China, and the results showed that the new-generation suffered from more depressive symptoms than the old-generation. At the same time, many studies [12–15] had explored depressive symptoms among elderly migrants (aged 60 and above) who migrated due to grandchild’s care, better health care services and family care, but their original intention was different from that of older rural-to-urban migrant workers. However, the potential effects on migration of older rural-to-urban migrant workers’ mental health status has rarely been empirically recognized. In addition, few evidence was available to evaluate the potential effects of migration on older rural-to-urban migrant workers’ mental health.

Limit studies on older rural-to-urban migrant workers in mainland China has primarily focused on their health; however, research regarding the comparison between older rural-to-urban migrant workers in mainland China and their rural and urban counterparts is scarce. To our best of knowledge, only Dan et al. had specially focused on the health of older rural-to-urban migrant workers in mainland China, and their study showed that older rural-to-urban migrant workers had higher self-assessed health status, compared with older rural dwellers [16]. A growing number of comparative studies on depressive symptoms between rural-urban migrants verse urban and rural counterparts had presented a conflicting picture. For example, Li et al. [17] discovered that rural-to-urban migrant workers suffered from worse depressive symptoms than their counterparts in the rural and urban areas. Zhong et al. [18] undertook a meta-analysis, and reported that rural-to-urban migrant workers experienced a greater severity of depressive symptoms than the general population. Li et al. [11] concluded that rural-to-urban migrant workers suffered from less depressive symptoms than their urban counterparts. Very few related studies [19] had specially focused on depressive symptoms of older rural-to-urban migrant workers in mainland China. Min et al. explored how social support affected older rural-to-urban migrant workers’ mental health, and it has been reported that social support had a significant effect on their mental health. However, there is a lack of comparison of depressive symptoms between older rural-to-urban migrant workers and their rural and urban counterparts, and the explanations for the differences have been less explored, not mentioning the probable determinants of the differences.

Social-Ecological Model provided a good framework to identify factors influencing the outcome at various levels [20]. Various studies have been conducted on how factors at multiple levels of the Social-Ecological Model contribute to depressive symptoms [21–25]. Social-Ecological Model contextualizes individuals’ behaviors using dimensions including biological characteristics and perceived susceptibility, life-style and health behavior, social interpersonal, living and working conditions; and social system, to provide a framework for describing the interactions between five spheres [20].

Although there is increasing recognition of older rural-to-urban migrant workers in mainland China, their mental health evaluation associated with migration is still in its infancy. As there were no consistency conclusions about mental health mechanisms about older rural-to-urban migrant workers. Our paper aimed to show the differences in depressive symptoms at multiple levels of the Social-Ecological Model between older rural-to-urban migrant workers versus older rural dwellers, and between older rural-to-urban migrant workers versus older urban residents. Therefore, based on the Social-Ecological Model, our study aimed to reveal the differences in depressive symptoms between older rural-to-urban migrant workers and older rural dwellers, and the differences between older rural-to-urban migrant workers and older urban residents. Our study further quantified the impact of biological characteristics and perceived susceptibility, life-style and health behavior, social interpersonal, living and working conditions; and social system on the depressive symptoms of older rural-to-urban migrant workers and their urban and rural counterparts. Then Fairlie’s decomposition was applied to deeply analyze the source of the differences in depressive symptoms. Accordingly, we provided insights into the issues of depressive symptoms among older rural-to-urban migrant workers, to deepen our understanding of this vulnerable group in mainland China.

## Methods

### Data

We adopted a dataset from Chinese Health and Retirement Longitudinal Study (CHARLS) conducted in 2015, and the data was issued by the China Center for Economic Research at Beijing University. By using the PPS Method (Probability Proportional to Size), `28 provinces in mainland China were randomly selected in the first stage, and 150 county-level units were selected in the next stage. Thirdly, 450 village-level units were chosen. Then, 12,400 households were interviewed. Finally, 23,000 individuals who were aged 45 and older were interviewed. These samples can be tracked every two to 3 years in the future, and a detailed description of the questionnaire had been published [26]. The CHARLS study has get the approval for interviewing respondents and collecting data by the Biomedical Ethics Review Committee of Peking University, and the informed consent was required to sign by the respondents.

In our study, older rural-to-urban migrant workers included met the following inclusion criteria: (1) aged 50 ~ 65; (2) with rural hukou (Chinese household registration system); (3) their permanent was cities and towns; (4) employed for more than 6 months. Older rural dwellers included met the following inclusion criteria: (1) aged 50 ~ 65; (2) with rural hukou; (3) their permanent was village; (4) not employed, or employed for less than 6 months. Older urban residents included met the following inclusion criteria: (1) aged 50 ~ 65; (2) with urban hukou; (3) their permanent was cities and towns. In our study, we restricted the age from 50 to 65 [27], to eliminate those who have exited the labor market. Four thousand eight hundred respondents (213 older rural-to-urban migrant workers, 3264 older rural dwellers and 703 older urban residents) were identified in the final sample for further analysis after data cleaning.

### Measurement

For the response variables of depressive symptoms, a Chinese version 10-item short form of the Center for Epidemiologic Studies-Depression Scale (CES-D 10) had exhibited a good internal consistent reliability and good construct (Cronbach’s alpha coefficients =0.813) [28, 29]. Per standard practice, the two positively oriented items, happiness and hope, were re-coded to be similar to the negatively oriented items. We used a 4 ~ point rating, ranging from rarely or none of the time (< 1 day), some or a little of the time (1 ~ 2 days), occasionally or a moderate amount of the time (3 ~ 4 days), to most or all of the time (5 ~ 7 days). Overall depressive symptoms was computed by combining the CES-D 10 values of ten items, which ranged from 0 to 30, with higher scores indicating more perceived poorer depressive symptoms. According to previous studies [28, 29], a score of 10 and over on the CES-D 10 indicated having depressive symptoms. Thus, the depression symptoms was a dummy variable equal to 1 if the score was 10 and over, and 0 otherwise.

### Social-ecological model

In our study, we followed the construction of some variables in previous research on ecological models of mental health [23, 24, 30–32]. According to the Social-Ecological Model, five dimensions and independent variables in our study can be constructed from the following aspects:

First, biological characteristics and perceived susceptibility: age group (50 ~ 60, 61 and above), gender (male, female), have you been ill in the last month (yes, no), self-reported health (completely satisfied, very satisfied, somewhat satisfied, not very satisfied, not at all satisfied) and chronic diseases (no, one, two and above).

Second, life-style and health behavior variables: smoking, drinking, sleeping time at night (<=4 h, 4 h ~ 8 h, > 8 h) and nap after lunch (yes, no).

Third, social interpersonal variables considered in the study were living arrangement (married with spouse present, live without spouse present), education level (below primary school, primary school, middle school and above) and social activity (none, one, two and above).

Four, living conditions and economic status: type of in-house shower (hot water provided, water heater installed by the household, no), clear in this house (good, fair, bad), geographic characteristics reflecting the potential regional heterogeneity (east, central, west) and expenditure. Expenditure was measured by yearly personal expenditure, and then was divided into five quintiles, from the poorest expenditures quintile and the richest quintile.

Five, social system considered in the study were type of health insurance {basic health insurance [including new cooperative medical insurance (NCMS [33])], no} and type of pension insurance [basic pension insurance, no] .

### Coarsened exact matching method

By using comparative analysis approach, our study analyzed the differences of depressive symptoms among older rural-to-urban migrant workers and their urban and rural counterparts. As Mark [34] put it, migration for work was not decided by a random selection. Contrarily, it involved several selections, such as self-selection and financial selection. To eliminate the deviation caused the selections and guarantee better balance of empirical distributions of the covariates between the comparison groups, we applied Coarsened exact matching (CEM) [35–38]. The approach helped to avoid the logic cycle in the matching process and reduce model dependence between the comparison groups. In general, the basic algorithm of CEM mainly included three procedures. The first step was to coarsen the variables to groups and appoint the indistinguishable values with the same value. Second, the algorithm of exact matching was employed. After removing the coarsened data, the final matched data should be reserved [39, 40]. In this study, groups were matched based on the employment status, so that they were comparable. In our study, migration for work in cities for older rural-to-urban migrant workers represented a different change in status. It meant “moving out” for older rural residents, but it meant “moving in” for older urban residents. Due to the Hukou system in mainland China, there was a huge gap between urban and rural residents, both in terms of socio-economic characteristics and mental health service. Therefore, we estimated the depressive symptoms in the three older groups by CEM. The covariate distributions of the data for those who moved out from rural areas and those who did not are different, and the covariate distributions for those who moved to cities and those who did not were also different. In our study, gender, age group, living arrangement, educational attainment, health insurance, pension insurance and economic status were used for the variable matched. If we simply put three categories together and use older rural-to-urban migrants as the reference group in same model, the variables matched would make the three older groups less comparable. Also, it might lead to the bias caused by the migration status(i.e., moving in or moving out). Therefore, we made two comparisons between older rural-to-urban migrant workers versus older rural dwellers, and between older rural-to-urban migrant workers versus older urban residents. In addition, CEM can improve estimates of the causal effect with the lowest bias for any sample size [41]. The increased efficiency and lower bias properties of CEM were attributed to stratification and exact matching of the treated and non-treated groups based on variables that explained variance in the outcome of our interest, difference-in-difference computations, and strata-based weighting within a nonparametric framework.

The multivariate imbalance measure *L*_1_ can be used to test the imbalance before and after CEM. *L*_1_ ranged from 0 to 1 (0 standed for perfect balance and 1 standed for maximal imbalance). A higher value meant a larger imbalance between comparison groups. A lower value meant more perfect global balance. A substantial reduction in *L*_1_ indicated a well-balanced matching [40]. If a sufficient degree of bias has been removed, the weights can be used in descriptive statistics and Logit models to determine the causal effect of the treatment effect [40]. Details on how to compute CEM in Stata can be found in previous studies [42]. CEM is an ado command by Blackwell, not an official Stata command, and CEM can be modeled by using the “cem” command code in Stata15.0.

### Decomposition method

Decomposition method was used to decompose the mental health differences into the contribution of various factors. If the mental health outcome was a continuous variable, Oaxaca-Blinder decomposition method was extensively adopted to analyze the contributions of health differences in different groups [43–45]. In most cases, however, the mental health outcome variables were seldom continuous. Since our outcome variables were dummy indicating whether the respondent currently suffered from depression symptoms, our study used the non-linear decomposition methods proposed by Fairlie and Bartus [46].

Following Fairlie [47], the decomposition for a nonlinear equation, $Y=F\left(X\overset{\wedge }{\beta}\right)$ can be written as:

1 $$ {\overline{\mathrm{Y}}}^{\mathrm{w}}\hbox{-} {\overline{\mathrm{Y}}}^{\mathrm{B}}=\left[\sum \limits_{i= 1}^{N^w}\frac{F\left({Xi}^w{\hat{\beta}}^w\right)}{N^w}\hbox{-} \sum \limits_{i= 1}^{N^B}\frac{F\left({Xi}^B\;{\hat{\beta}}^w\right)}{N^B}\right]+\left[\sum \limits_{i= 1}^{N^B}\frac{F\left({Xi}^B\;{\hat{\beta}}^w\right)}{N^B}\hbox{-} \sum \limits_{i= 1}^{N^B}\frac{F\left({Xi}^B\;{\hat{\beta}}^B\right)}{N^B}\right] $$

To calculate the decomposition, we defined $\overset{-w}{Y}$ and $\overset{-B}{Y}$ as the average probability of the binary mental health outcomes of two groups, and F as the cumulative distribution function from the logistic distribution. $\overset{-w}{Y}-\overset{-B}{Y}$ represented the total gap due to group differences. Where N^*j*^ was the sample size for group j. In our study, j presented these two groups of w and B. This alternative expression for the decomposition was used because Y did not necessarily equal. $F{\left(X\overset{\wedge }{\beta}\right)}^3$ The equation showed that the differences was made up of two components: explained component and unexplained component. In (1), the first term in brackets represented the part of the gap that was due to group differences in observed characteristics and a part attributable to differences in the estimated coefficients. The second term represented the part because of the differences caused by the levels of Y. Contribution to the differences in depressive symptoms between different older groups and the proportion of contribution in the differences were reported.

### Statistical analysis

The descriptive statistics analyses showed the details, and the chi-square test was used to examine categorical variables. The logistic regression was applied with weighted data to estimate the association between influencing factor and depressive symptoms. Multicollinearity was quantified by variable inflation factors (VIF); and the cut-offs of 5, 10, and sometimes 30 would indicate problematic levels of multicollinearity [43]. All results were presented as odds ratios with 95% confidence intervals (CIs). Finally, Fairlie’s decomposition was performed for the contributions of the differences. All procedures were conducted using STATA 15.0 (StataCorp LP., College Station, TX, USA). The statistical significance level was defined as 0.05.

## Result

Tables 1 and 2 showed summary statistics for the characteristics of respondents before and after matching. It was obvious that there were statistically significant differences on some characteristics between older rural-to-urban migrant workers and older rural dwellers, and between older rural-to-urban migrant workers and older urban residents before matching. Therefore, to compare their depressive symptoms, we need to match key variables by CEM to control confounding factors. It was obvious that there was almost no statistically significant difference on these characteristic after matching, which indicated good matching performances, and thus these groups became more comparable. According to Tables 1 and 2, older rural-to-urban migrant workers reported a lower prevalence of depressive symptoms than older rural dwellers, but a higher prevalence of depressive symptoms than older urban residents.

**Table 1:** Variables for older rural-to-urban migrant workers and older rural dwellers before after matching

	Before Matching N(%)			After Matching N(%)		
	Older rural-to-urban migrant workers	Older rural dwellers	*p*-value	Older rural-to-urban migrant workers	Older rural dwellers	*p*-value#
Depressive symptoms			< 0.05			0.092
Yes	167(23.76)	1239(37.96)		55(30.73)	839(38.31)	
No	536(76.24)	2025(62.04)		124(69.27)	1351(61.69)	
**Biological characteristics & perceived susceptibility variables**						
Gender			0.144			1.000
Men^a^	122(57.28)	1701(52.11)		105(58.66)	1701(52.11)	
Women	91(42.72)	4862(45.95)		74(41.34)	1563(47.89)	
Age group			< 0.05			1.000
50–60 ^a^	153(71.83)	1455(66.44)		127(70.95)	2113(64.74)	
61 and above	60(28.17)	735(33.56)		52(29.05)	1151(35.26)	
Ill in the last month			0.158			0.306
Yes^a^	19(8.92)	270(12.33)		17(9.5)	397(12.16)	
No	194(91.08)	1920(87.67)		162(90.50)	2867(87.84)	
Self-reported health			0.325			0.979
Completely satisfied^a^	9(4.23)	70(3.2)		7(3.91)	117(3.58)	
Very satisfied	39(18.31)	452(20.64)		32(17.88)	704(21.57)	
Somewhat satisfied	108(50.70)	1021(46.62)		88(49.16)	1489(45.62)	
Not very satisfied	46(21.60)	468(21.37)		42(23.46)	687(21.05)	
Not at all satisfied	11(5.16)	179(8.17)		10(5.59)	267(8.18)	
Chronic diseases			0.082			0.388
No^a^	93(43.66)	835(38.13)		77(43.02)	1296(39.71)	
Only one	49(23.00)	667(30.46)		44(24.58)	987(30.24)	
Two and above	71(33.33)	688(31.42)		58(32.40)	981(30.06)	
**Life-style and health behavior variables**						
Smoke			0.487			0.463
Still smoking^a^	57(31.84)	1051(32.2)		62(29.11)	747(34.11)	
Quit	116(64.80)	2123(65.04)		143(67.14)	1383(63.15)	
Never smoked	6(3.35)	90(2.76)		8(3.76)	60(2.74)	
Drink (in the past year)			0.718			0.687
Drink more than once a month^a^	58(32.40)	935(28.65)		66(30.99)	643(29.36)	
Drink but less than once a month	15(8.38)	293(8.98)		20(9.39)	199(9.09)	
None of these	106(59.22)	2036(62.38)		127(59.62)	1348(61.55)	
Sleeping time			< 0.05			0.178
< =4^a^	32(17.88)	541(16.57)		37(17.37)	353(16.12)	
4 ~ 8	136(75.98)	2390(73.22)		165(77.46)	1621(74.02)	
> 8	11(6.15)	333(10.2)		11(5.16)	216(9.86)	
Nap			0.218			0.439
Yes^a^	110(61.45)	1867(57.2)		131(61.50)	1250(57.08)	
No	69(38.55)	1397(42.8)		82(38.50)	940(42.92)	
**Social interpersonal variables**						
Living arrangement			0.082			1.000
Live with spouse^a^	176(82.63)	2538(77.76)		148(82.68)	1868(85.3)	
Live without spouse	37(17.37)	726(22.24)		31(17.32)	322(14.7)	
Educational attainment			0.096			0.616
Primary school and above ^a^	174(81.69)	363(11.12)		16(8.94)	256(11.69)	
Primary school	24(11.27)	2468(75.61)		140(78.21)	1823(83.24)	
Middle school and above	15(7.04)	433(13.27)		23(12.85)	111(5.07)	
Social activity			< 0.05			< 0.05
None^a^	64(30.05)	1489(45.62)		56(31.28)	1033(47.17)	
1	70(32.86)	966(29.6)		63(35.20)	619(28.26)	
> =2	79(37.09)	809(24.79)		60(33.52)	538(24.57)	
**Living conditions and economic status**						< 0.001
Running water			< 0.001			
Yes^a^	190(89.20)	2291(70.19)		158(88.27)	1514(69.13)	
No	23(10.80)	973(29.81)		21(11.73)	676(30.87)	
Type of in-house shower						
Hot water provided^a^	6(2.82)	16(0.49)	< 0.001	4(2.23)	12(0.55)	< 0.05
Water heater installed by the household	157(73.71)	1740(53.31)		132(73.74)	1132(51.69)	
No	50(23.47)	1508(46.2)		43(24.02)	1046(47.76)	
Clear in this house			< 0.001			0.256
Good^a^	82(38.50)	964(29.53)		65(36.31)	638(29.13)	
Fair	110(51.64)	1973(60.45)		95(53.07)	1352(61.74)	
Bad	21(9.86)	327(10.02)		19(10.61)	200(9.13)	
Region			0.261			0.972
East^a^	80(37.56)	1275(39.06)		69(38.55)	817(37.31)	
Middle	87(40.85)	1164(35.66)		70(39.11)	804(36.71)	
West	46(21.60)	825(25.28)		40(22.35)	569(25.98)	
Quintiles			0.371			1.000
Poorest^a^	34(15.96)	688(21.08)		30(16.76)	376(17.17)	
Poorer	50(23.47)	653(20.01)		42(23.46)	444(20.27)	
Middle	46(21.60)	640(19.61)		40(22.35)	488(22.28)	
Richer	41(19.25)	662(20.28)		34(18.99)	492(22.47)	
Richest	42(19.72)	621(19.03)		33(18.44)	390(17.81)	
**Social system**						
Health insurance			< 0.001			1.000
Basic health insurance^a^	186(87.32)	3110(95.28)		186(87.32)	2161(98.68)	
None	27(12.68)	154(4.72)		27(12.68)	29(1.32)	
Pension insurance			0.499			1.000
Basic health insurance^a^	155(72.77)	2443(74.85)		155(72.77)	1759(80.32)	
No	58(27.23)	821(25.15)		58(27.23)	431(19.68)	
N	213	3477		179	2369	

^a^Reference levels in the regressions; Virtual variables for chi-2 test; *p*#-value indicated the weight to be considered

**Table 2:** Variables for older rural-to-urban migrant workers and older urban residents before after matching

Dependent variables	Before Matching N (%)			After Matching N (%)		
	Older rural-to-urban migrant workers	Older urban residents	*p*-value	Older rural-to-urban migrant workers	Older urban residents	*p*-value#
Depressive symptoms			0.191			0.077
Yes	60(28.17)	167(23.76)		55(28.50)	97(22.00)	
No	153(71.83)	536(76.24)		138(71.50)	344(78.00)	
**Biological characteristics & perceived susceptibility variables**						
Gender			0.144			1.000
Men^a^	122(57.28)	457(65.01)		114(59.07)	248(56.24)	
Women	91(42.72)	246(34.99)		79(40.93)	193(43.76)	
Age group			< 0.05			1.000
50–60 ^a^	153(71.83)	338(48.08)		141(73.06)	320(72.56)	
61 and above	60(28.17)	365(51.92)		52(26.94)	121(27.44)	
Ill in the last month			0.158			0.806
Yes^a^	19(8.92)	68(9.67)		17(8.81)	42(9.52)	
No	194(91.08)	635(90.33)		176(91.19)	399(90.48)	
Self-reported health			0.325			0.929
Completely satisfied^a^	9(4.23)	25(3.56)		9(4.66)	19(4.31)	
Very satisfied	39(18.31)	102(14.51)		35(18.13)	60(13.61)	
Somewhat satisfied	108(50.70)	413(58.75)		96(49.74)	261(59.18)	
Not very satisfied	46(21.60)	132(18.78)		43(22.28)	82(18.59)	
Not at all satisfied	11(5.16)	31(4.41)		10(5.18)	19(4.31)	
Chronic diseases			0.082			1.000
No^a^	93(43.66)	255(36.27)		86(44.56)	86(44.56)	
Only one	49(23.00)	189(26.88)		42(21.76)	42(21.76)	
Two and above	71(33.33)	259(36.84)		65(33.68)	65(33.68)	
**Life-style and health behavior variables**						
Smoke			0.487			0.341
Still smoking^a^	57(31.84)	183(26.03)		56(29.02)	123(27.89)	
Quit	116(64.80)	496(70.55)		130(67.36)	298(67.57)	
Never smoked	6(3.35)	24(3.41)		7(3.63)	20(4.54)	
Drink (in the past year)			0.718			0.628
Drink more than once a month^a^	58(32.40)	215(30.58)		62(32.12)	140(31.75)	
Drink but less than once a month	15(8.38)	87(12.38)		17(8.81)	56(12.70)	
None of these	106(59.22)	401(57.04)		114(59.07)	245(55.56)	
Sleeping time			< 0.05			0.242
< =4^a^	32(17.88)	81(11.52)		31(16.06)	44(9.98)	
4 ~ 8	136(75.98)	593(84.35)		151(78.24)	380(86.17)	
> 8	11(6.15)	29(4.13)		11(5.70)	17(3.85)	
Nap			0.218			0.281
Yes^a^	110(61.45)	417(59.32)		119(61.66)	259(58.73)	
No	69(38.55)	286(40.68)		74(38.34)	182(41.27)	
**Social interpersonal variables**						
Living arrangement			0.082			1.000
Live with spouse^a^	176(82.63)	576(81.93)		166(86.01)	395(89.57)	
Live without spouse	37(17.37)	127(18.07)		27(13.99)	46(10.43)	
Educational attainment			0.096			1.000
Primary school and above ^a^	174(81.69)	30(4.27)		8(4.15)	8(1.81)	
Primary school	24(11.27)	482(68.56)		36(18.65)	339(76.87)	
Middle school and above	15(7.04)	191(27.17)		111(5.07)	94(21.32)	
Social activity			< 0.05			0.175
None^a^	64(30.05)	187(26.60)		58(30.05)	112(25.40)	
1	70(32.86)	173(24.61)		61(31.61)	119(26.98)	
> =2	79(37.09)	343(48.79)		74(38.34)	210(47.62)	
**Living conditions and economic status**						< 0.05
Running water			< 0.001			
Yes^a^	190(89.20)	674(95.87)		171(88.60)	419(95.01)	
No	23(10.80)	29(4.13)		22(11.40)	22(4.99)	
Type of in-house shower						
Hot water provided^a^	6(2.82)	10(1.42)	< 0.001	4(2.07)	6(1.36)	0.895
Water heater installed by the household	157(73.71)	527(74.96)		143(74.09)	333(75.51)	
No	50(23.47)	166(23.61)		46(23.83)	102(23.13)	
Clear in this house			< 0.001			0.111
Good^a^	82(38.50)	316(44.95)		78(40.41)	213(48.30)	
Fair	110(51.64)	324(46.09)		98(50.78)	187(42.40)	
Bad	21(9.86)	63(8.96)		17(8.81)	41(9.30)	
Region			0.261			0.740
East^a^	80(37.56)	232(33.00)		73(37.82)	145(32.88)	
Middle	87(40.85)	368(52.35)		80(41.45)	236(53.51)	
West	46(21.60)	103(14.65)		40(20.73)	40(20.73)	
Quintiles			0.371			1.000
Poorest^a^	34(15.96)	118(16.79)		26(13.47)	52(11.79)	
Poorer	50(23.47)	142(20.20)		45(23.32)	95(21.54)	
Middle	46(21.60)	152(21.62)		44(22.80)	100(22.68)	
Richer	41(19.25)	142(20.20)		38(19.69)	104(23.58)	
Richest	42(19.72)	21.19(21.19)		40(20.73)	90(20.41)	
**Social system**						
Health insurance			< 0.001			1.000
Basic health insurance^a^	186(87.32)	583(82.93)		172(89.12)	398(90.25)	
None	27(12.68)	120(17.07)		21(10.88)	43(9.75)	
Pension insurance			0.499			1.000
Basic health insurance^a^	155(72.77)	476(67.71)		143(74.09)	329(74.60)	
No	58(27.23)	227(32.29)		50(25.91)	112(25.40)	
N	213	703		193	441	

^a^Reference levels in the regressions; Virtual variables for chi-2 test; *p*#-value indicated the weight to be considered

Table 3 showed the multivariate *L*_1_ statistics. After matching, *L*_1_ between older rural-to-urban migrant workers and older rural dwellers was actually close to zero, which was much lower than that before matching (0.478), indicating good matching performances; *L*_1_ between older rural-to-urban migrant workers and older urban residents was all actually close to zero, which was much lower than that before matching (0.456), which also indicated good matching performances.

**Table 3:** *L*_1_ measure of imbalance before and after Coarsened Exact Matching

Variables	Before Matching		After Matching		Before Matching		After Matching	
	older rural-to-urban migrant workers		older rural dwellers		older rural-to-urban migrant workers		older urban residents	
	L_1_	mean	L_1_	mean	L_1_	mean	L_1_	mean
Age group	0.052	−0.052	2.3e-15	9.5e-15	0.052	−0.052	3.3e-16	−2.4e-15
Gender	0.071	−0.071	2.5e-15	−2.7e-15	0.071	−0.071	3.1e-16	−8.9e-16
Living arrangement	0.049	−0.049	6.5e-16	2.7e-15	0.049	−0.049	1.2e-16	2.2e-15
Educational attainment	0.050	0.078	5.1e-16	6.7e-16	0.050	0.078	1.2e-16	−3.1e-15
Social activity	0.175	−0.086	1.0e-15	3.6e-14	0.175	−0.086	1.8e-16	−8.9e-16
Smoke	0.164	−0.069	2.7e-15	1.8e-15	0.164	−0.069	2.5e−16	-1.3e15
Drink	0.062	0.071	3.5e-15	8.4e-15	0.062	0.071	5.8e-16	−7.1e-15
Income quintiles	0.052	−0.052	2.3e-15	9.5e-15	0.052	−0.052	3.3e-16	−2.4e-15
L_1_	0.478		3.4e-15		0.456		5.9e-16	

*L*_1_ reported the *L*_1*j*_ measure, which is *L*_1_ computed for the j th variable separated. The mean was labeled in parentheses reported the difference in means

Table 4 showed that, in the comparison of older rural-to-urban migrant workers and older urban residents, correlates for depressive symptoms of older rural-to-urban migrant workers included age, self-reported health and sleeping time; while correlates for older rural dwellers included gender, ill in the last month, self-reported health, chronic diseases, sleeping time and type of in-house shower. In the comparison of older rural-to-urban migrant workers and older urban residents, correlates for depressive symptoms of older rural-to-urban migrant workers included age group, self-reported health, sleeping time and clear in this house; while correlates for older rural dwellers included age, self-reported health, sleeping time, social activity and region.

**Table 4:** Association of independent variables and depressive symptoms in matched multivariate regression analysis

	Older rural-to-urban migrant workers			Older rural dwellers			Older rural-to-urban migrant workers			Older urban residents		
	Odds Ratio	[95% CI]		Odds Ratio	[95% CI]		Odds Ratio	[95% CI]		Odds Ratio	[95% CI]	
Gender	0.856	−0.019	1.731	−0.114***	− 0.325	0.096	1.020	0.136	1.903	−0.807	− 1.472	− 0.142
Age group	0.654*	− 0.389	1.697	0.566	0.322	0.810	0.948*	− 0.090	1.986	0.473*	−0.178	1.124
Ill in the last month	0.111	−1.254	1.477	−0.551***	− 0.837	− 0.264	0.097	− 1.331	1.525	0.225	− 0.636	1.086
Self-reported health	1.044***	0.487	1.600	0.600***	0.489	0.712	1.069***	0.519	1.620	1.086***	0.700	1.472
Chronic diseases	0.031	−0.474	0.537	0.266***	0.147	0.385	−0.155	−0.657	0.346	0.297	−0.023	0.616
Smoke	−0.586	−1.553	0.381	−0.080	−0.294	0.135	−0.384	−1.349	0.582	−0.075	− 0.629	0.479
Drink	0.164	−0.381	0.710	0.076	−0.049	0.200	0.133	−0.404	0.669	−0.015	−0.374	0.345
Sleeping time	−0.944*	−1.810	−0.078	− 0.611***	−0.806	− 0.416	−1.001*	− 1.891	−0.112	− 0.943**	−1.610	−0.276
Nap	0.693	−0.118	1.503	0.076	−0.119	0.271	0.316	−0.475	1.107	0.302	−0.226	0.830
Living arrangement	0.710	−0.315	1.735	0.245	−0.030	0.520	0.743	−0.320	1.806	0.383	−0.419	1.186
Educational attainment	0.429	−0.451	1.309	−0.010	−0.253	0.234	−0.465	−1.416	0.486	−0.063	− 0.679	0.552
Social activity	−0.459	−0.972	0.054	−0.100	− 0.219	0.020	− 0.351	−0.833	0.132	−0.344*	− 0.670	−0.018
Running water	−0.204	−1.419	1.011	0.098	−0.113	0.309	−0.028	−1.232	1.176	−1.013	−2.304	0.279
Type of in-house shower	−0.429	−1.356	0.499	0.483***	0.286	0.680	−0.539	−1.456	0.378	0.303	−0.287	0.893
Clear in this house	0.561	−0.094	1.215	0.160	−0.007	0.327	0.756*	0.100	1.411	0.044	−0.360	0.448
Region	0.266	−0.284	0.815	0.186	0.062	0.310	0.306	−0.256	0.867	0.538*	0.123	0.952
Income quintiles	0.111	−0.196	0.418	0.007	−0.067	0.082	0.168	−0.142	0.477	−0.105	−0.319	0.108
Health insurance	0.423	−0.933	1.779	−0.034	−0.887	0.820	−0.470	−1.959	1.019	−0.051	−1.009	0.907
pension insurance	0.334	−0.560	1.227	0.173	−0.070	0.415	0.386	−0.509	1.280	−0.006	−0.594	0.583

Confidence interval; * *p* < 0.05, ** *p* < 0.01, *** *p* < 0.001. We found low VIFs, < 10, reflecting no multicollinearity problem in the logistic regressions; all predictors entered the multivariate regression simultaneously

Table 5 further showed how the determinants influenced the differences in depressive symptoms between older rural-to-urban migrant workers and older rural dwellers, and between older rural-to-urban migrant workers and older urban residents. 54.15% of differences between older rural-to-urban migrant workers and older rural dwellers under the Social-Ecological Model would be explained; only 20.56% of differences between older rural-to-urban migrant workers and older urban residents would be explained. Our findings confirmed that type of in-house shower (84.21%), sleeping time (− 15.77%) and ill in the last month (7.09%) were highly significant in explaining Gap (1); while self-reported health (− 49.65%) and sleeping time (− 49.00%) were highly significant in explaining Gap (2).

**Table 5:** Fairlie’s decomposition of depressive symptoms difference

	Gap (1)			Gap(2)		
Total gap	0.0631			0.0653		
Explained (%)	0.0342 (54.15%)			0.0134 (20.56%)		
Explained						
Contribution to difference	Contribution (%)	[95% CI]		Contribution (%)	[95% CI]	
Gender	−0.44	− 0.002	0.001	− 0.08	− 0.003	0.003
Age group	−9.09	− 0.007	0.001	− 57.40	− 0.018	0.002
Ill in the last month	7.09*	0.000	0.004	2.14	−0.003	0.003
Self-reported health	−6.15	−0.005	0.003	−49.65*	−0.014	−0.001
Chronic diseases	0.21	−0.001	0.001	3.26	−0.003	0.004
Smoke	1.59	−0.002	0.003	−16.79	−0.007	0.002
Drink	−0.39	−0.002	0.001	−4.93	−0.005	0.004
Sleeping time	−15.77***	−0.009	−0.002	−49.00*	− 0.013	−0.004
Nap	2.47	−0.001	0.003	26.62	−0.002	0.009
Living arrangement	1.17	−0.001	0.002	6.65	−0.003	0.005
Educational attainment	0.00	−0.001	0.001	−2.05	−0.003	0.003
Social activity	18.67	−0.002	0.014	13.88	−0.003	0.006
Running water	5.78	−0.008	0.012	47.77	−0.003	0.016
Type of in-house shower	84.21***	0.016	0.042	2.09	−0.006	0.003
Clear in this house	2.81	−0.002	0.004	−19.31	−0.007	0.002
Region	2.64	0.007	0.032	−3.18	−0.004	0.003
Income quintiles	0.22	−0.001	0.002	−1.16	−0.004	0.003
Health insurance	0.66	−0.002	0.002	3.61	−0.003	0.004
Pension insurance	−0.52	−0.002	0.001	−2.24	−0.003	0.003

Gap (1): older rural-to-urban migrant workers and older rural dwellers; Gap (2): older rural-to-urban migrant workers and older urban residents; *CI* Confidence interval; * *p* < 0.05, *** *p* < 0.001

## Discussion

To the best of our knowledge, this was the first large-scale comparative study in mainland China in depressive symptoms between older rural-to-urban migrant workers versus older urban residents, and between older rural-to-urban migrant workers versus older urban residents. The issue of older rural-to-urban migrant workers in mainland China has attracted widespread attention among the whole society [1]. Our study tried to measure differences in depressive symptoms between older rural-to-urban migrant workers and their rural and urban counterparts in mainland China. CEM helped reduce bias in the data, allowing for a more accurate and stronger further analysis. Our study confirmed that there were indeed differences in depressive symptoms between older rural-to-urban migrant workers and their rural and urban counterparts.

After matching, we found a lower prevalence of depressive symptoms in older rural-to-urban migrant workers than older rural dwellers, and a higher prevalence of depressive symptoms in older rural-to-urban migrant workers than older urban residents. Older rural-to-urban migrant workers had lower prevalence of depressive symptoms than older rural dwellers, in agreement with previous studies on “healthy migrant hypothesis” [48]. It may be due to the fact that older rural-to-urban migrant workers typically left their homes to improve employment opportunities and economic status, which is favorable for improving depressive symptoms. By contrast, in line with some findings [49], the disadvantage of depressive symptoms in older rural-to-urban migrant workers versus older urban residents was founded in our study. Several reasons could partially explain it as follows. First, older rural-to-urban migrant workers without a city “hukou” faced substantial barriers of entry to employment in some major industries and corporations, and many of them were forced to accept jobs with unfairness and low pay. Second, older rural-to-urban migrant workers in cities were more likely to have poor living conditions [50, 51], and be isolated in social networks [52]. Unfortunately, although Chinese government had taken different measures to help older rural-to-urban migrant workers to settle down in cities, there is very little attention paid to differences in depressive symptoms of older rural-to-urban migrant workers and older urban residents.

Our regression analyses further showed differentials in correlates of depressive symptoms among older rural-to-urban migrants, older rural dwellers and older urban residents in mainland China. As highlighted by other researchers, age, sleeping time, gender, ill in the last month, chronic diseases, type of in-house shower, clear in this house, social activity and region were key indicators for depressive symptoms [53–55]. While no significant associations between personal expenditure and region were found. Self-reported health and sleeping time exerted obvious impacts on depressive symptoms among older rural-urban migrants and their urban and rural counterparts. That was, the older groups who had worse self-reported health and less sleeping time at night were more likely to suffer from depressive symptoms. It was suggested that different strategies should be adopted, considering the heterogeneity of different older groups.

Our results suggested that there were strong differences in depressive symptoms between older rural-urban migrants verse urban and rural counterparts. Depressive symptoms detrimental to older rural-urban migrants was clearly observable here for both base populations verse urban counterparts. It’s probably because older rural-urban migrants had lower socio-economic position, and poorer living conditions in the city. However, their mental health advantage stood out when they were compared with their rural counterparts. Improved economic conditions experienced by older rural-urban migrants might explain their better mental health relative to their rural counterparts. Though continuous progress has been made in mental health services in mainland China, a huge gap in depressive symptoms remained wide between older rural-to-urban migrant workers and their counterparts.

Our study provided convincing evidence that priority should be given to improving depressive symptoms for older rural-to-urban migrant workers. A large proportion of the differences still cannot be explained by the observed differences. On one hand, the differences already seem to reveal that some discrimination was taking place, particularly the large gap between older rural-to-urban migrant workers and older urban residents. Owing to the unobservability of social policies and social integration, we can’t estimate the influence of social policies and cultural factors in our study. It reminded us of the need to reduce discrimination in policy setting and social integration. On the other hand, although variables had been included as many as possible in our study, our study can’t include all factors that influence depression caused by data limitations. Our study did not make a detailed introduction about the unexplained part of the decomposition in lines with other studies [56–58], because the unexplained part was partly captured differences in unmeasured characteristics, and it was sensitive to the choice of left-out categories, making the results difficult to interpret.

The most important contribution of our study did not only evaluate risk factors for depressive symptoms among older rural-to-urban migrant workers, older rural dwellers and older urban residents, but also explored the explanatory source of the differences in depressive symptoms by Fairlie’s decomposition analysis. As many studies had mentioned, the significance of sleep loss and sleep deficiency were drivers of depressive symptoms [59, 60], and our results further suggested that sleeping time at night was important contributory factors for the differences of depressive symptoms between older rural-urban migrants verse urban and rural counterparts. Longer sleeping time can improve depressive symptoms as observed in our study. Our study identified that sleeping time at night decreased differences in depressive symptoms between older rural-urban migrants verse urban and rural counterparts. Intervention programs on sleep deprivation and depression should be activated by targeting different groups.

Our study also reminded that type of in-house shower was a key contributor to eliminate the potential differences in depressive symptoms between older rural-to-urban migrant workers and older rural dwellers. Many older rural dwellers can’t take a warm bath or shower, because of the lack of hot bath facilities, so we stressed the importance of mainland China’s rural residential solar hot water heating system programs. Given that ill in the last month and self-reported health appeared to be important contributors for differences in depressive symptoms between older rural-to-urban migrant workers and older urban residents, it was suggested that physical health was an important factor for depressive symptoms. Therefore, it is very essential to recognize different risk-factor profiles of different older groups and make different policy approaches on depressive symptoms.

Although a substantial amount of evidence had investigated associations between economy status and depressive symptoms [61, 62], our result found that no statistically significant differences in depressive symptoms were identified in living expenses. It may be due to the fact that older population in mainland China often gave their money to their left-behind family members, or they accumulated money for later, instead of using money to promote mental well-being. Our studies would further assess the relationship between living expenses and depressive symptoms.

Several limitations still required our attention. First, because of the cross-sectional design of our study, our study cannot provide causal inferences. We can get a very small sample size if we matched the data from 2011 to 2015. Therefore, we didn’t use the panel data of CHARLS. The aging of older rural-to-urban migrant workers is a dynamic process, and a longitudinal study using other data should be investigated in our future research to emphasize causal relationships. Second, the unexplained part from decomposition analysis still accounted for a big percentage of total differences. This unexplained part revealed that some discrimination was taking place, and it may be due to the contribution by unobserved variables such as social norms and culture. More factors should be put into the model in our further study, to warrant a better understanding of differences in depressive symptoms. Third, older rural-to-urban migrant workers were highly selected sample groups, and small sample sizes meant that some estimates lacked precision. Pooled estimates were used to address it; however, it may mask heterogeneity between groups. Although CEM was more effective and convenient than tendency score matching method, the poor representativeness of the study population still existed [63]. Finally, we can’t identify urban-urban migrants and rural-rural migrants in our analyses. More comprehensive studies are warranted to explore these groups.

## Conclusion

The comparative analysis showed that significant differences in depressive symptoms between older rural-urban migrants verse urban and rural counterparts did exist. Moreover, we also found that type of in-house shower, sleeping time, ill in the last month and self-reported health were key factors of differences in depressive symptoms. Based on the contributing factors which may result in the differences in depressive symptoms, more efforts are urged to improve the mental health condition of the older rural-urban migrants. Therefore, Chinese government should adopt countermeasures to improve depressive symptoms and narrow differences in depressive symptoms to achieve a stable and sustainable urbanization process.

## Data Availability

CHARLS is a large-scale interdisciplinary survey project hosted by the National Development Research Institute of Peking University. The datasets analysed during the current study are available in the CHARLS, [http://charls.pku.edu.cn/en].

## Abbreviations

NBC: National Bureau of Statistics
CHARLS: China Health and Retirement Longitudinal Study
PPS: Probability Proportional to Size
CEM: Coarsened exact matching method
CES-D 10: Chinese version 10-item short form of the Center for Epidemiologic Studies-Depression Scale
NCMS: New cooperative medical insurance
hukou: Chinese household registration system
WHO-5: World Health Organization Five-item Well-Being
SF-36: The MOS item short from health survey
VIF: Variable inflation factors
